# Small-molecule degron mimetics for targeted protein degradation

**DOI:** 10.1042/EBC20253026

**Published:** 2025-10-22

**Authors:** Xingui Liu

**Affiliations:** Department of Medicinal Chemistry, College of Pharmacy, University of Florida, FL32610, U.S.A.

**Keywords:** degron, degron mimetics, N-degron, C-degron, hydroxyproline degron, cyclic imide degron, phosphodegron

## Abstract

More than 80% of intracellular proteins are degraded by the ubiquitin-proteasome system. This system relies on a cascade of enzymes—E1 (ubiquitin-activating enzyme), E2 (ubiquitin-conjugating enzyme), and E3 (ubiquitin ligase)—to catalyze the polyubiquitination of target proteins, which are then recognized and degraded by the 26S proteasome. Among these enzymes, E3 ubiquitin ligases play a central role by specifically recognizing degron motifs on substrate proteins. The presence and accessibility of these degrons often dictate the half-life and stability of a given protein. Leveraging this mechanism, the artificial introduction of degrons or degron mimetics into otherwise stable proteins has emerged as a novel strategy in drug discovery for selectively degrading disease-causing proteins. In this short review, I will highlight small-molecule degron mimetics that have been developed for targeted protein degradation.

## Introduction

**Figure 1:**
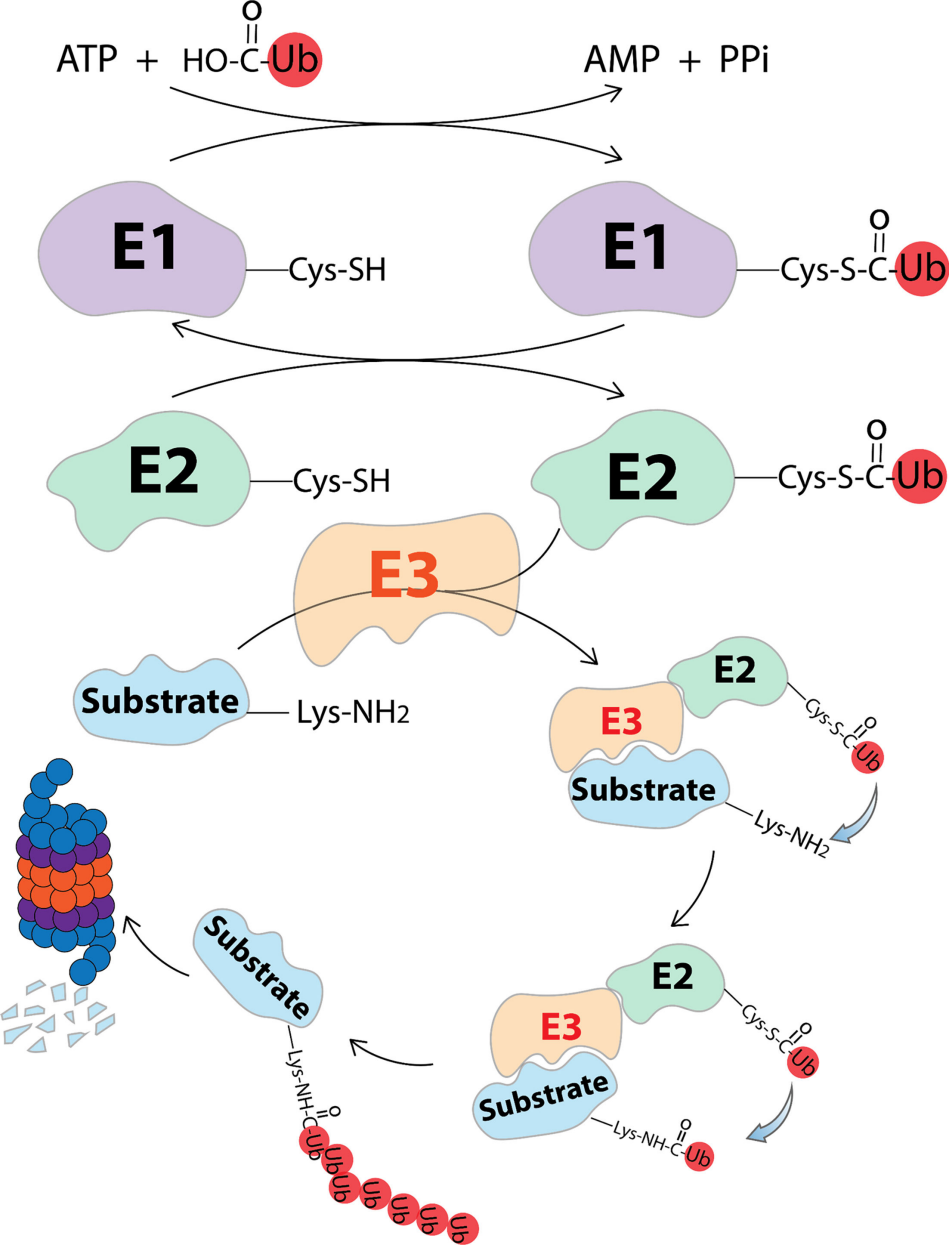
Ubiquitin-proteasome degradation pathway orchestrated by E1, E2, E3, and the proteasome. E1 is responsible for activating the ubiquitin molecule by forming a high-energy thioester bond with the C-terminal cysteine residue of ubiquitin. The activated ubiquitin was then transferred to E2 through a trans-thioesterification reaction. Finally, E3 recruits the substrate and guides the transfer of the ubiquitin from the E2 active site cysteine to the ϵ-amine of a lysine residue on the substrate. After multiple cycles of ubiquitination, the polyubiquitinated substrate is recognized and degraded by proteasome

Ubiquitin-proteasome system (UPS) and lysosome pathways are the two key systems responsible for protein degradation [1,2]. While the lysosome pathways can degrade extracellular, membrane-bound proteins and protein aggregates, the turnover of more than 80% of intracellular proteins is mediated by the UPS [3–5]. The UPS utilizes an orchestrated enzymatic cascade of E1, E2, and E3 ligases to attach ubiquitin to the target protein, resulting in the degradation of the polyubiquitinated target protein by the 26S proteasome [4,6] (Figure 1). This degradation pathway is a highly regulated and specific process, ensuring only the intended proteins are degraded. The specificity of the process is conferred by the interactions between E3 ubiquitin ligase and degradation signals, also known as degrons on the target protein [4].

**Figure 2:**
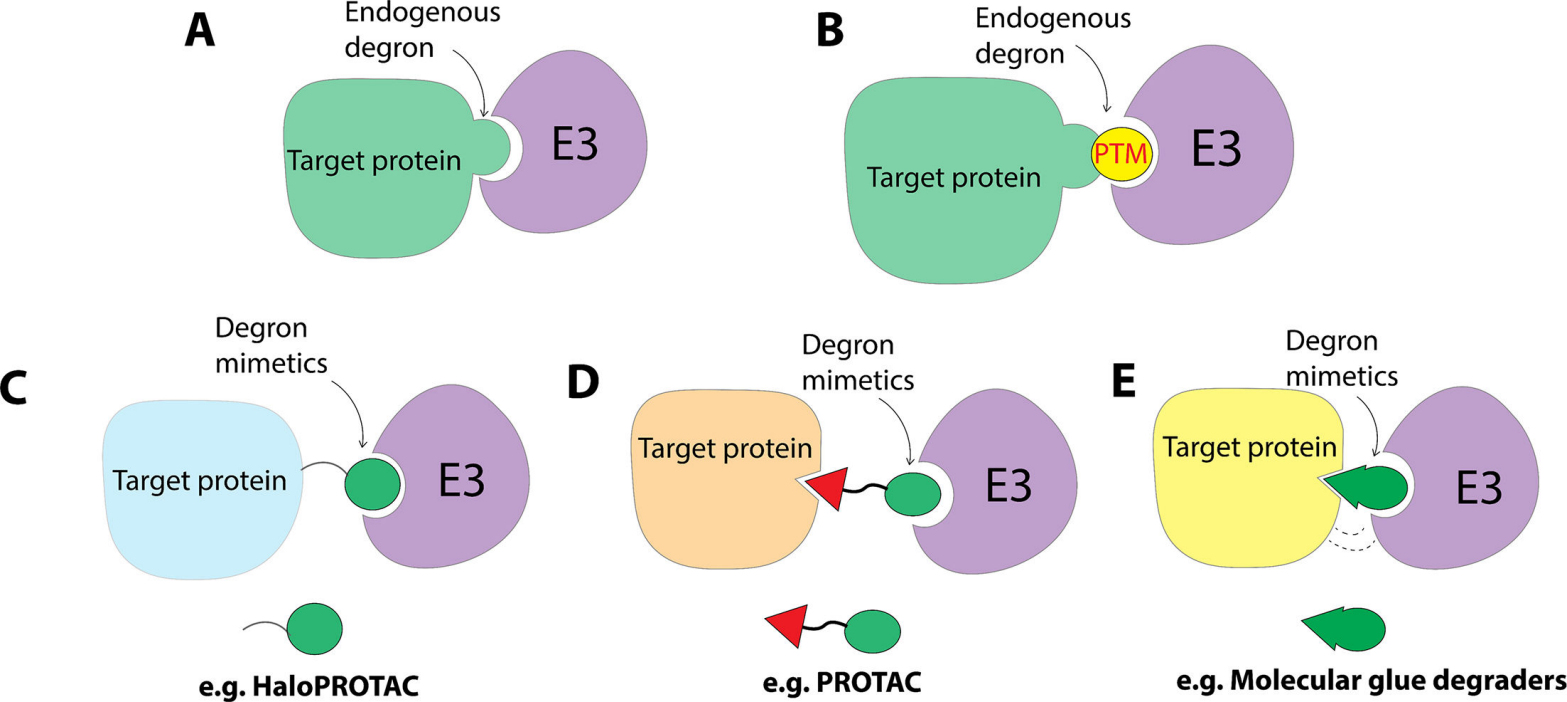
E3 ligase recognizes degron and degron mimetics on target protein. Degron mimetics can be introduced to target protein in the form of covalent tag (e.g. HaloPROTAC), non-covalent bifunctional molecules (e.g. PROTAC), and monovalent molecule glue degraders. PTM, post-translational modification; PROTAC, proteolysis-targeting chimeras.

A degron is defined as the minimal element within a protein that is sufficient for recognition and degradation by a proteolytic apparatus [7,8] (Figure 2). The degron can be short amino acid sequences, short structural motifs located anywhere within the protein, and exposed amino acids (such as lysine and proline), which are often found at the terminal site of a protein [9] (Figure 2A). In addition to the constitutional degrons encoded within a protein’s gene, degrons can also be introduced to a protein in a controlled manner through post-translational modifications (PTMs) (Figure 2B), such as phosphorylation, hydroxylation, glycosylation, and acetylation [7,10,11]. Artificially transferring degrons to an otherwise stable protein enables its recognition by E3 ligases, leading to subsequent polyubiquitination and degradation [12]. This degron transferability laid the foundation for small-molecule-induced targeted protein degradation (TPD), where degron mimetics are introduced to target proteins via a variety of ways [13] (Figure 2). For example, HaloPROTACs (HaloTag-based Proteolysis Targeting Chimeras) can covalently introduce degron mimetics to HaloTag fusion proteins, enabling the recruitment of E3 ligases and subsequent degradation of the fusion proteins (Figure 2C). Proteolysis-targeting chimeras (PROTACs) use heterobifunctional structures to noncovalently attach degron mimetics to target proteins. One end of the molecule binds the target protein, while the other end contains a degron mimetic (e.g. hydroxyproline-based mimetics), connected via a chemical linker (Figure 2D). Molecular glue degraders, in contrast, are monovalent small molecules that function as degron mimetics at the interface between the target protein and the E3 ligase (Figure 2E). In this review, we will focus on small-molecule degron mimetics that have been developed for TPD.

## N-degrons and N-degron mimetics

First discovered in 1986, N-degron pathway [14] has been shown to be responsible for the selective degradation of proteins bearing destabilizing N-terminal motifs (*aka* N-degrons). N-degrons are initially cryptic as proN-degrons and can be exposed through enzymatic cleavage. Enzymes that are involved in exposing N-degrons include caspases [15,16], calpains [17], separases [18], cathepsins [19], ribosome-associated methionine aminopeptidases (MetAPs) and other non-MetAP aminopeptidases [20,21]. Upon exposure, the N-degron can be recognized by specific E3 ligases [15–21] or other proteins (e.g. p62/SQSTM1 [22]), resulting in the protein degradation by the UPS or lysosome system.

**Figure 3:**
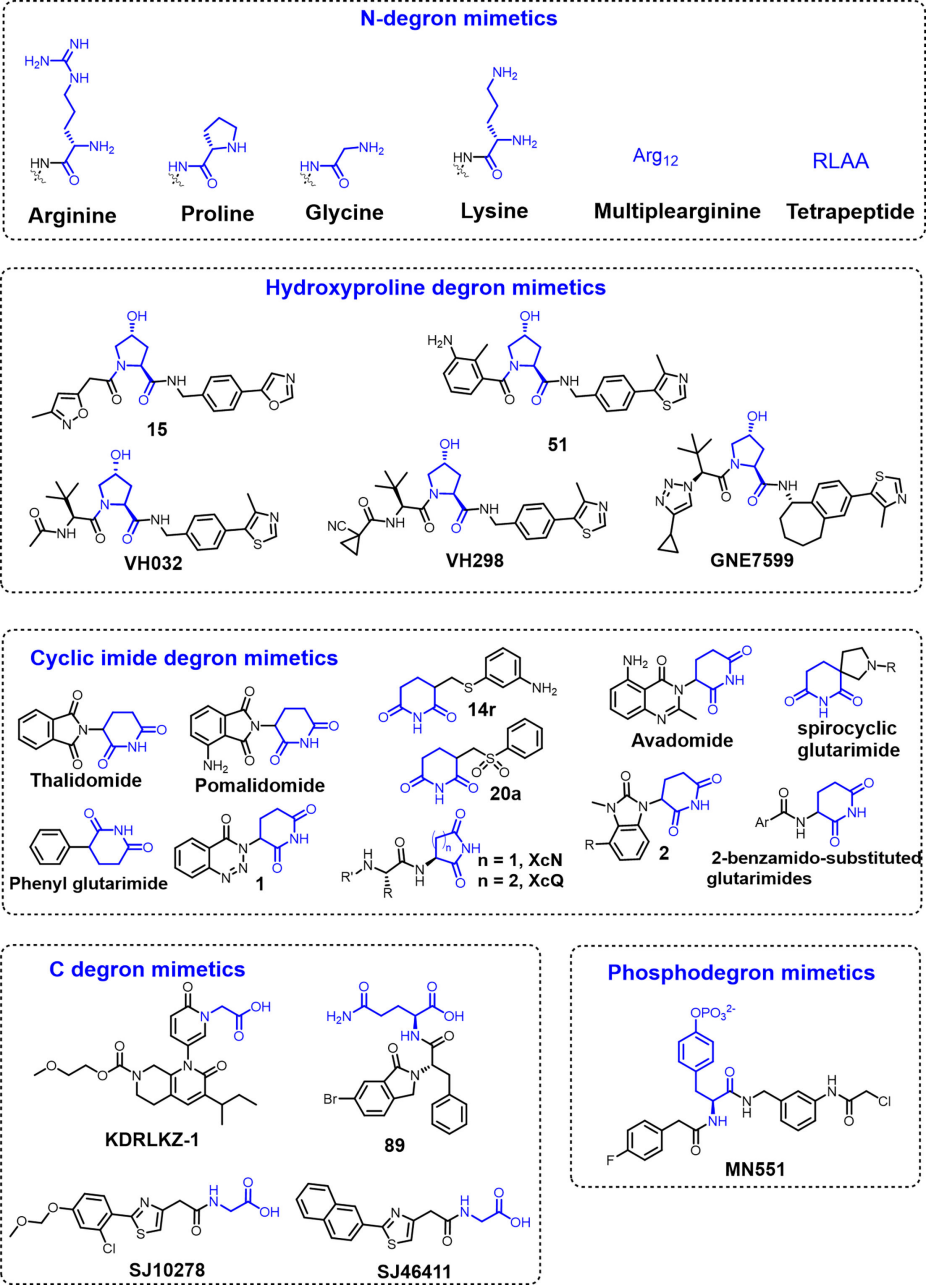
Structures of representative degron mimetics.

All 20 amino acids can function as destabilizing N-terminal residues under certain conditions [23], underlying their potential in the design of small-molecule protein degraders. Using Gly, Pro, and Lys as N-degrons (Figure 3), Zhang et al. conjugated these amino acids with kinase inhibitors and developed amino acid-based proteolysis-targeting chimeras (AATacs). These AATacs recruit ZYG11B/ZER1, GID4, or UBR1/2/4 E3 ligase to induce the degradation of kinases such as chinoderm Microtubule-Associated Protein-Like 4 (EML4-ALK) and Epidermal Growth Factor Receptor (EGFR) [24,25]. Similarly, the same group demonstrated that arginine-based AATacs could degrade BCR-ABL effectively both *in vitro* and *in vivo* [26]. One notable advantage of AATacs is that it eliminates the need to preselect a specific E3 ligase for TPD. Instead, the most compatible N-degron recognizing E3 ligase can be recruited, even if its identity is explicitly unknown. Besides single amino acids, poly-arginine chains [27] and tetrapeptides such as RLAA (Figure 3) [28,29] have also been used as N-degron mimetics for TPD.

In addition to directly attaching N-degrons to target proteins to induce their degradation, alternative strategies for harnessing the N-degron pathway in TPD could include hijacking enzymes responsible for N-terminal exposure. For instance, methionine aminopeptidases (MetAPs) can expose native N-degrons by cleaving the initiator methionine, while arginyl-tRNA–protein transferase 1 (ATE1) [30] can conjugate N-degron arginine to the N-terminus of target proteins. Bifunctional molecules capable of inducing the proximity between target proteins and these N-terminal exposing enzymes represent a promising direction for expanding the scope of N-degron-based TPD.

### Hydroxyproline degron mimetics

Hydroxylation, particularly of proline residues, is a PTM that can mark protein for degradation via the UPS [31,32]. It is involved in regulating the homeostasis of many proteins, including β2-adrenergic receptor [33], N-myc downstream-regulated gene 3 (NDRG3) [34], Filamin A (FLNA) [35] and the most well-known hypoxia-inducible factor 1α (HIF-1α) [36–38]. Under normoxic conditions, these proteins undergo prolyl hydroxylation, which facilitates their recognition by the von Hippel-Lindau (VHL) E3 ligase, leading to polyubiquitination and degradation via the UPS. In contrast, hypoxia or inhibition of prolyl hydroxylation prevents their degradation, resulting in stabilization [31,32].

Given hydroxyproline’s critical role in facilitating substrate recognition by the VHL E3 ligase, Buckley et al. employed a fragment-growing approach to develop hydroxyproline analogs aimed at disrupting the interaction between VHL and its primary substrate, HIF-1α [39,40]. These hydroxyproline derivatives (e.g. **15** and **51**) (Figure 3) mimic hydroxylated HIF-1α in binding to VHL and can function as degron mimetics when attached to target proteins that are not naturally recognized by VHL. Further structure-guided modifications by the Ciulli group and others led to the identification of several potent hydroxyproline-based degron mimetics including VH032, VH298 [41,42], GNE7599 [43], and many others [44,45]. These hydroxyproline degron mimetics are widely used in PROTACs design, making VHL the second-most harnessed E3 ligase for TPD [46]. Interestingly, with small structural changes to known hydroxyproline degron mimetics, these molecules can also be turned into molecular glue degraders, inducing the degradation of neo-substrates (e.g. cysteine dioxygenase 1 [47] and GEMIN3 [48]) by reprogramming VHL’s substrate recognition interface. While a specific N-degron could potentially be recognized by multiple E3 ligases, the recognition of hydroxyproline degron is thus far limited to VHL.

### Cyclic imide degron mimetics

Cereblon (CRBN) is a crucial substrate recognition adapter within the Cullin-Ring E3 ubiquitin ligase 4 complex, which comprises DDB1, CUL4, and RBX1 [49]. Thalidomide and its analogs, pomalidomide and lenalidomide, collectively referred to as immunomodulatory drugs (IMiDs) [50], bind to CRBN and modulate its substrate specificity. Upon binding, IMiDs facilitate the recruitment of neo-substrates, such as IKZF1/3, CK1α, and SALL4 [51–53], promoting their polyubiquitination and subsequent proteasomal degradation. This mechanism underlies both the therapeutic efficacy of IMiDs in diseases like multiple myeloma and del(5q) myelodysplastic syndrome, and their teratogenicity [52–58]. Despite safety concerns, the strong and selective CRBN binding by IMiDs has led to their widespread use in designing PROTACs [59–61] and molecular glue degraders [62,63], making CRBN the most commonly recruited E3 ligase in TPD [64]. CRBN-based degraders have successfully targeted a broad range of proteins, from kinases and cellular receptors to transcription factors, cytokines, and anti-apoptotic proteins [61], demonstrating their versatility. Notably, most CRBN ligands, including IMiDs, share a glutarimide structure moiety, which is believed to be essential for CRBN engagement [55,65,66]. According to the studies by Ichikawa et al., this glutarimide group mimics cyclic imides degrons—naturally occurring motifs recognized by CRBN [58,67]. Endogenous cyclic imide degrons can arise through post-translational cyclization of glutamine or asparagine residues [58]. A variety of synthetic cyclic imide degron mimetics have been developed (Figure 3), with applications in both molecular glue discovery and PROTAC design [65,68,69].

### C-degron mimetics

The C-degron pathway, as the name indicates, targets proteins for degradation based on specific amino acid sequences at their C-terminal which are also known as C-degrons. A diverse array of E3 ligases has been found to regulate their substrates’ stability through C-degron recognition [12,70–76]. All these E3 ligases employ tandem repeat domains, such as Kelch repeats, tetratricopeptide repeats, and Ankyrin repeats, to facilitate degron recognition [77].

The Kelch domain-containing protein 2 (KLHDC2), KLHDC3, KLHDC10, and KLHDC1 are well-characterized E3 ligases recognizing distinct G-end motifs in substrates, with KLHDC2 and KLHDC1 showing preference for -GG, KLHDC3 targeting -RG and -KG, while KLHDC10 recognizing -AG, -WG, and -PG [12,78,79]. KDRLKZ-1 is the first small-molecule mimetic of the diglycine C-degron discovered through computational *de novo* small-molecule discovery (Figure 3). Structurally, KDRLKZ-1 kept the terminal glycine but replaced the n-1 glycine with a pyridinone ring [80]. Independent research from the Schulman group reported the discovery of a new type of diglycine C-degron mimetic (e.g. SJ10278 and SJ46411) that bind to KLHDC2 with micromolar or sub-micromolar binding affinities (Figure 3) [81]. Both types of diglycine C-degron mimetics have been successfully incorporated into PROTAC design for TPD [80,81]. Tripartite motif-containing 7 (TRIM7) is a ubiquitin E3 ligase that specifically recognizes degron sequences containing C-terminal glutamine via its PRYSPRY domain [72,82,83]. By constructing a library of peptide ligands that mimic the φGln degron found in TRIM7 substrates, Knapp’s group identified compound 89 as a TRIM7-binding ligand (Figure 3) [84]. One of the key challenges associated with C-degron mimetics is the presence of a carboxylate moiety, which is typically essential for engaging the E3 ligase but hinders cellular permeability. To address this limitation, ester-based prodrug strategies have been employed to enhance membrane penetration [80,81,84]. It would be interesting to explore whether other medicinal chemistry strategies, such as bioisosteres substitution, could serve as alternative approaches to overcoming the permeability issue.

## Phosphodegron mimetics

Protein phosphorylation at the serine, threonine, or tyrosine residues within the regulatory domains or degron regions of target proteins is one of the most important PTMs for regulating protein stability [11,85]. Phospho-inactivated degrons lose their binding affinities to substrate-recognizing domain of E3 ligase following phosphorylation, leading to protein stabilization [86–88]. A good example is the DNA replication inhibitor geminin, which is phosphorylated on its D box degron motif by Aurora Kinase A. The phosphorylation diminishes its affinity to anaphase-promoting complex/cyclosome (APC/C) E3 ligase and therefore inactivates the degron. Only after the phosphorylation is removed can geminin be degraded by APC/C [86]. In contrast, phospho-activated degrons have increased affinity with the degron-recognizing subunits of E3 ligase upon phosphorylation, resulting in more efficient substrate degradation [89]. For example, Cyclin E is recognized and ubiquitinated by Skp1-Cullin1-F-box E3 ligase only after phosphorylation at S384 by cyclin-dependent kinase 2 (CDK2) and T380 by glycogen synthase kinase-3 [90,91]. Similarly, double-phosphorylation of β-catenin at S37 and S33 forms the phospho-activated degron motif that is recognized by β-transducin repeats-containing proteins (β-TrCP), leading to ubiquitination and destruction of β-catenin [92].

Since a specific phosphomotif on substrate proteins serves as a docking site for E3 ligase, phosphomotif mimetics can be developed as E3 ligands [93]. The Src homology 2 (SH2) domain of the E3 ligase suppressor of cytokine signaling 2 (SOCS2) recognizes phosphorylated tyrosine residues on several substrates, including the growth hormone receptor (GHR)-pY595, erythropoietin receptor (EpoR)-pY426 [94] and leptin receptor (pY1077) [94], thereby regulating their turnover. Leveraging key interactions between the SH2 domain of SOCS2 and phosphorylated peptides derived from GHR-pY595 and EpoR-pY426, Ramachandran et al. discovered a tyrosine phosphomimetic molecule MN551 (Figure 3) using a structure-guided design combined with a fragment-based growing approach [93]. However, due to the negatively charged phosphate group, MN551 exhibits poor cell permeability, although it can engage with SOCS2 protein effectively. To overcome this limitation, a prodrug strategy was employed to facilitate intracellular delivery. Further efforts to incorporate this tyrosine mimetic into PROTACs design are still ongoing [93]. As an alternative to phosphodegron mimetics development, *in situ* generation of endogenous phospho-activated degrons using phosphorylation-inducing chimeric small molecules (PHICS) [95,96] also presents a promising strategy for TPD.

## Conclusion

**Figure 4:**
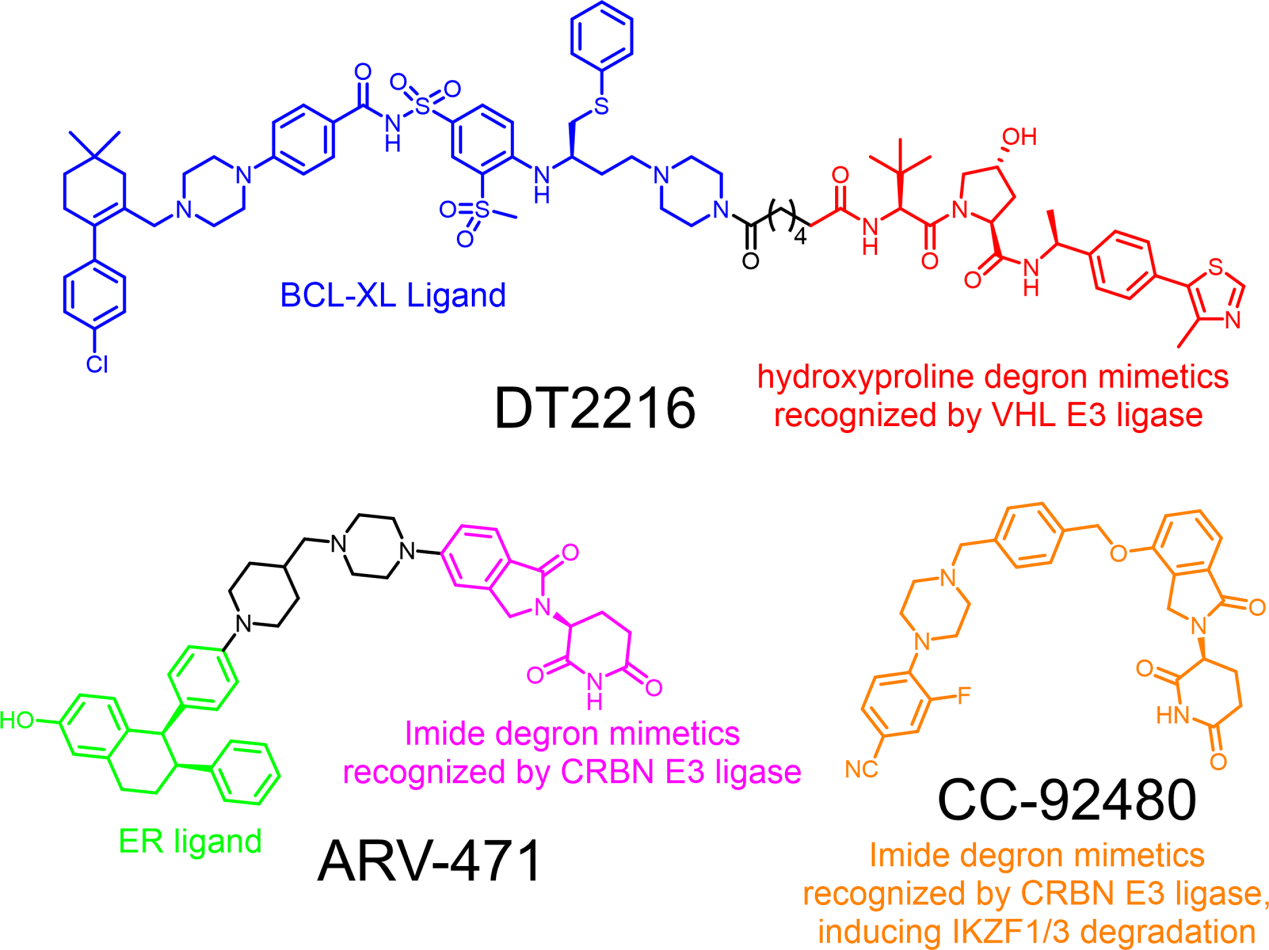
Successful examples of utilizing degron mimetics in the design of protein degraders. BCL-XL: B-cell lymphoma-extra large; VHL: Von Hippel-Lindau; CRBN: cereblon; ER: estrogen receptor; IKZF1/3: IKAROS Family Zinc Finger 1/3.

TPD has emerged as a promising strategy in drug discovery, with PROTACs and molecular glue degraders representing the two predominant modalities. Both approaches function by simultaneously engaging a target protein and an E3 ligase, leading to target polyubiquitination and subsequent degradation by the proteasome. However, due to the limited availability of E3 ligase ligands, current PROTAC and molecular glue degraders rely on only a handful of E3 ligases, out of around 600 human E3 ligases, for target protein degradation [97]. Expanding the toolbox of E3 ligase ligands is becoming an actively pursued research area, not only to expand the chemical space and broaden the degradable target space of protein degraders but also to overcome drug resistance and minimize on-target and off-target toxicity of current degraders [98–100]. Multiple strategies have been employed to identify new ligands, including *in silico* drug screening [80,101], high-throughput compound library screening [102], fragment-based drug discovery [103–105], DNA-encoded library screening [105], and chemical proteomic approach [106–109]. In addition, leveraging specific interactions between degrons and E3 ligases presents an attractive avenue for E3 ligand development. Degron mimetics, which mimic the natural recognition elements of E3 ligases, can serve as viable ligands in the design of degraders, as demonstrated by several successful examples current in clinical trials (Figure 4). This review summarizes representative small-molecule degron mimetics, such as N-degron mimetics, C-degron mimetics, hydroxyproline degron mimetics, cyclic imide degron mimetics, and phosphodegron mimetics. With the continued identification of novel degrons and E3 substrate recognition motifs, the development of new degron mimetics is expected to further diversify the toolbox for TPD.

**Summary**

E3 ligases recognize endogenous substrates through specific degron motifs. Degron motifs are modular and transferable, small-molecule degron mimetics that can be appended to neo-substrates to induce their degradation. Degron mimicry is a viable approach for recruiting E3 ligase for targeted protein degradation purposes.

## Abbreviations

EEGSK-3: glycogen synthase kinase-3
AATacs: amino acid-based proteolysis-targeting chimeras
APC/C: anaphase-promoting complex/cyclosome
ATE1: arginyl-tRNA-protein transferase 1
CDK2: cyclin-dependent kinase 2
CRBN: cereblon
EML4-ALK: chinoderm Microtubule-Associated Protein-Like 4
ER: estrogen receptor
EpoR: erythropoietin receptor
FLNA: Filamin A
GHR: growth hormone receptor
HIF-1α: hypoxia-inducible factor 1 α
HaloPROTACs: HaloTag-based Proteolysis Targeting Chimeras
IKZF1/3: IKAROS Family Zinc Finger 1/3
IMiDs: immunomodulatory drugs
KLHDC2: Kelch domain-containing protein 2
MetAPs: methionine aminopeptidases
NDRG3: N-myc downstream-regulated gene 3
PHICS: phosphorylation-inducing chimeric small molecules
PTM: post-translational modification
SH2: Src homology 2
SOCS2: suppressor of cytokine signaling 2
TPD: targeted protein degradation
TRIM7: tripartite motif containing 7
UPS: ubiquitin-proteasome system
VHL: von Hippel-Lindau
β-TrCP: β-transducin repeats-containing proteins
